# Wanted, Lady Mission Doctors

**Published:** 1908-02-08

**Authors:** H. H. Montgomery


### WANTED, LADY MISSION DOCTORS.

Sir,—I thankfully inform you that the immediate vacancy at Delhi caused by the death of Dr. Marie Hayes has been filled by one who has formerly had experience in that mission. It was an excellent offer; we dismissed her with blessing to-day, and she sails for India next week. At the same time, the appeal which you so kindly admitted to your columns still remains in force, as the work cannot be fully equipped without another qualified woman on the staff, and the other vacancies alluded to still remain.

H. H. Montgomery, Bishop.

